# Biological Features of the Outcome-Based Intrinsic Capacity Composite Scores From a Population-Based Cohort Study: Pas de Deux of Biological and Functional Aging

**DOI:** 10.3389/fmed.2022.851882

**Published:** 2022-03-04

**Authors:** Lin-Chieh Meng, Shih-Tsung Huang, Li-Ning Peng, Liang-Kung Chen, Fei-Yuan Hsiao

**Affiliations:** ^1^Graduate Institute of Clinical Pharmacy, College of Medicine, National Taiwan University, Taipei, Taiwan; ^2^Aging and Health Research Center, National Yang Ming Chiao Tung University Yangming Campus, Taipei, Taiwan; ^3^Center for Geriatrics and Gerontology, Taipei Veterans General Hospital, Taipei, Taiwan; ^4^Taipei Municipal Gan-Dau Hospital, Taipei, Taiwan; ^5^School of Pharmacy, College of Medicine, National Taiwan University, Taipei, Taiwan; ^6^Department of Pharmacy, National Taiwan University Hospital, Taipei, Taiwan

**Keywords:** intrinsic capacity, biomarkers, genetic markers, mortality, healthy aging

## Abstract

**Introduction:**

This study aims to develop and validate an integrative intrinsic capacity (IC) scoring system, to investigate its associations with a wide spectrum of biomarkers and to explore the predictive value of the integrative IC score on 4-year mortality among community dwelling people aged 50 years and older.

**Methods:**

We included 839 adults aged ≥50 years from the Social Environment and Biomarkers of Aging Study (SEBAS) and randomly divided them into derivation and validation cohorts to develop the IC scoring system. The multivariate logistic regression model was used to weight each subdomain (locomotion, sensory, vitality, psychological, and cognition) of IC according to its association with impairments in instrumental activities of daily living (IADL) and to construct the integrative IC score. Age-related biomarkers and genetic markers were compared between IC groups by ordinal logistic regression. A Cox proportional hazard model was used to examine the association between IC and mortality, and subgroup analysis was used to assess the robustness of the results among participants aged 60 years and older.

**Results:**

A 12-score IC scoring system (AUROC = 0.83; Hosmer–Lemeshow goodness-of-fit test *p* = 0.17) was developed, and higher scores indicated better intrinsic capacity. High interleukin (IL)-6, high E-selectin, low serum albumin and low folate were significantly associated with low IC in the whole sample. However, high IL-6, low serum albumin, low folate, high allostatic load, and APOE ε4 genotype were significantly associated with low IC in those aged 60 years old and older. Compared to the high IC group, the low IC group was significantly associated with all-cause mortality (HR: 2.50, 95% CI: 1.22–5.11, *p* = 0.01 for all participants; HR 2.19, 95% CI 1.03–4.64, *p* = 0.04 for participants aged 60 years and older).

**Conclusions:**

The conceptually proposed IC can be easily transformed into a scoring system considering different weights of individual subdomains, which not only predicts mortality but also suggests different pathophysiologies across the life course of aging (inflammation, nutrition, stress, and ApoE4 genotype). An intervention study is needed using the composite IC score to promote healthy aging and determine the underlying pathophysiology.

## Introduction

The World Health Organization (WHO) published the World Report on Aging and Health and highlighted the importance of intrinsic capacity and functional ability to promote healthy aging, which shifted the focus of health care systems from a disease-centric to a function-centric approach. The intrinsic capacity (IC), defined as the composite of all physical and mental capacities of an individual, is therefore proposed to also serve as a potential indicator of functional reserve in the aging process. According to the conceptual proposal, IC consists of five pivotal domains, i.e., locomotion, sensory, vitality, psychological, and cognition (1–4). Through interactions with environmental factors, IC affects different dimensions of functional ability over time. The Integrated Care for Older People (ICOPE) guidelines published by the WHO emphasize the importance of assessment, prevention, and development of personalized intervention strategies for IC declines (5), which is especially critical to promote healthy aging. Although the conceptual framework and dimensions of IC are generally agreed upon, no universally agreed operational definition of IC has been established. A previous study in nursing home residents indicated that certain individual domains of IC (i.e., balance and nutrition) significantly predicted the adverse outcomes of mortality and risk of falling (6). Other studies constructed their own IC scales and reported associations between IC, functional impairments and frailty (7, 8). However, most studies assigned equal weightage to each domain of IC (9, 10) premised on the assumption of equal contribution of each domain to outcomes, which can be problematic when developing a composite IC scale covering all components.

Although IC was conceptualized through a function-centric approach, the underlying biological features of IC remained unclear. Allostatic load, a model of biological aging, effectively captured the risk of adverse outcomes as people age and was inversely associated with IC (11). Moreover, biomarkers of systematic inflammation, e.g., homocysteine and C-reactive protein, have been reported to be associated with muscle weakness, slow gait, reduced physical function, and low IC (12–15). On the other hand, apolipoprotein E (ApoE) genotypes (16) and the serotonin transporter promotor polymorphism (5-HTTLPR) (17) strongly affect mental health and vitality in the construction of IC. Obviously, IC defined by functional phenotypic features may consist of intertwined biological features and their interactions. Until now, little was known about the biological features of IC, and the search for underlying biological features may further facilitate the development of a systematic approach linking age-related biological features and functional phenotypes.

Hence, the aims of this study are to develop and validate a composite IC scoring system that weights each subdomain according to its association with the functional outcome, to explore the underlying biological features of IC and to explore the predictive value of the integrative IC score on 4-year mortality in a nationwide population-based cohort study.

## Methods

### Data Source

This retrospective observational study used data from the Social Environment and Biomarkers of Aging Study (SEBAS), a national population-based cohort sample subsampled from the Taiwan Longitudinal Study of Aging (TLSA), to represent all Taiwanese individuals aged 50 years and older. TLSA has been conducted by the Health Promotion Administration (HPA) through face-to-face interviews since 1989 and was a national longitudinal survey designed to gather comprehensive data on demographics, socioeconomic factors, physical capacity, mental status and laboratory tests of study participants. SEBAS was approved by the institutional review board at the National Institute of Family Planning in Taiwan. The details of SEBAS and TLSA are described on the HPA website and in previous studies (18, 19).

### Study Population

Among 1,284 participants who joined the 2006 SEBAS survey, 1,036 participants who completed the health examination were retrieved for the present study. To capture the effects of IC during the natural aging process, participants aged 50 years and older were identified. Participants (*n* = 197) without available data on IC development were excluded, so a total of 839 study subjects were included in this study.

### Phase 1: Development of Intrinsic Capacity Score

The IC score was constructed according to the definition proposed by the WHO ICOPE (5). All components emerging from the guidelines and available in the SEBAS were selected. Each subdomain was divided into two categories and further weighted by their associations with their impairments in instrumental activities of daily living (IADL) measured in SEBAS 2006 survey, which indicated early loss of functional ability.

#### Locomotion Domain

The usual gait speed test and repeated chair-stand test were used to assess locomotion capacity. Robust was defined as walking speed or chair stand speed above the lowest quintile, and the slowest 20% of the population was defined as slowness (20).

#### Sensory Domain

The sensory domain was measured by self-reported hearing loss and visual impairments. Visual screening was performed using Snellen chart and was recorded by the decimal scale (i.e., 1.0 = 20/20). Visual impairment was defined as visual acuity of 6/18 or <6/12 (21).

#### Vitality Domain

Two variables were used for this domain: body mass index (BMI) and handgrip strength. The study subjects who were underweight, overweight, or obese (e.g., BMI <18.5 or ≥25.0) were considered potentially malnourished according to the WHO's definition (22, 23). Handgrip strength was measured by the North Coast™ hydraulic hand-dynamometer (NC70142, California, US), and the maximal reading of three trials was recorded. Weakness was defined by the Asian Working Group on Sarcopenia (AWGS) <28 kg for men and <18 kg for women (24), whereas others were recorded as robust.

#### Psychological Domain

The psychological domain was assessed by the 10-item Center for Epidemiologic Studies Depression (CES-D-10) for depressive symptoms and the 10-item Perceived Stress Scale (PSS-10) for stress. The cut-off point for defined depression was 8 or more on the CES-D-10 (25, 26); participants with a PSS-10 score ≥14 were considered to have higher levels of perceived stress (27, 28).

#### Cognition Domain

The Short Portable Mental Status Questionnaire (SPMSQ) (29), an easy-to-handle and validated tool, and two subparts of the Mini-Mental State Examination (MMSE) (30) related to language and 3-item recall were used to evaluate the cognitive performance of the study participants. Cognitive impairment was defined as an SPMSQ score ≥3 or lower than 1 standard deviation of the subdomains (language and 3-item recall) of the MMSE in the population.

#### Instrumental Activities of Daily Living Impairments

The 6-item Instrumental Activities of Daily Living (IADL), including purchasing personal items, managing money, taking bus/train alone, doing physical work at home, making telephone calls, and doing light tasks at home, was measured to assess the functional ability of our study participants. One or more impairments in IADL functions were defined as functional impairments.

### Phase 2: The Association Between Biomarkers and Intrinsic Capacity

All participants fasted for 10 h overnight, and then 12-h urine samples and venous blood samples were collected by the research staff. The samples were immediately shipped from the hospital to Union Clinical Laboratory (UCL) in Taipei by noon and were processed according to the standard laboratory protocol.

#### Biomarkers

As cardiometabolic risk markers, hemoglobin was measured by sodium lauryl sulfate (SLS) hemoglobin, and serum levels of homocysteine were assessed by using a fluorescence polarization immunoassay (FPIA, Abbott IMx). Hyperhomocysteinemia was defined as homocysteine concentrations >15 μmol/L (15). For neuroendocrine biomarkers, dehydroepiandrosterone sulfate (DHEA-S) was determined by electrochemiluminescence immunoassay (ECLIA, Roche Hitachi Elecsys 2010), and insulin-like growth factor-1 (IGF-1) was estimated by enzyme-linked immunosorbent assay (ELISA, Diagnostic System Laboratories).

Biomarkers related to inflammation and endothelial function, such as interleukin-6 (IL-6), soluble intercellular adhesion molecule-1 (s-ICAM-1), E-selectin, and soluble IL-6 receptor, were measured using ELISA (R&D Systems); fibrinogen was assessed by the coagulation method (Sysmex CA-1500; reagent: Dade Behring Company). In addition, serum albumin was measured, and a low serum albumin level (<4.0 g/dL) was defined according to previous research (31). As one of the B vitamins, folate was analyzed by chemiluminescence (Beckman CoulterACCESS^®^ Immunoassay analyser). To explore possible non-linear relationships, each biomarker was categorized into tertiles for analysis (Supplementary Table 1).

#### Allostatic Load

The selected biomarkers and cut-off points for AL construction were based on a previous study (32). In brief, 20 biomarkers related to cardiometabolism, neuroendocrine function, and inflammation were included in this study (Supplementary Table 2). For each biomarker, a value below the 10th and higher than the 90th percentile was considered positive for AL estimation, except for DHEA-S, high-density lipoprotein cholesterol (HDL-C), serum albumin, and serum creatinine. For DHEA-S, HDL-C, and serum albumin, a certain proportion of cortisol and epinephrine measurements were undetectable, and these undetectable values were categorized as positive for risk, which was consistent with a previous study (32).

#### Genetic Markers

To determine ApoE genotypes, DNA was extracted from whole blood and amplified using the polymerase chain reaction amplification refractory mutation system (PCR-ARMS) and polymerase chain reaction restriction fragment length polymorphism (PCR-RFLP) analysis. ApoE ε4 carriage was defined as having at least one ε4 allele. To determine the 5-HTTLPR genotype, DNA was extracted from venous blood and then amplified with polymerase chain reaction (PCR). Subjects were classified into four groups based on their genotypes (i.e., S/S, S/L, S/XL, and L/L or L/XL) (33).

### Phase 3: Intrinsic Capacity Predicts 4-Year All-Cause Mortality

#### Mortality

The follow-up status of all participants was obtained from their interview date until 31 December 2010, and the date of death was identified from the National Death Registry held by the Ministry of Health and Welfare, Taiwan, which was linked with the study database.

#### Other Variables

Demographic characteristics of all participants, including age, sex, and levels of education, were collected in SEBAS 2006 survey. Smoking status was defined as ever tobacco consumption in the past 6 months. A self-rated 10-score ladder scale was used to measure socioeconomic status. The number of comorbidities was documented according to self-reported physician diagnosis, including hypertension, diabetes, heart disease, stroke, cancer, pulmonary disease, gastric disease, liver disease, arthritis, kidney disease, gout, cataract, degenerative joint disease, spinal/vertebrae spur, and hip fracture.

### Statistical Analysis

#### Phase 1

In the first study phase, data from all participants were randomly divided into the derivation cohort (70%) and the validation cohort (30%) by sex and age. In the derivation cohort, univariate logistic regression was used to examine the associations between each IC component and IADL impairments. The significance level in univariate analyses was set at 0.25. Components with statistical significance or clinical importance in univariate analyses were selected for multivariate logistic regression. Although BMI was insignificant in univariate logistic regression, we kept it in the model as a clinical indicator of nutrition status. Subdomains with a *p-*value < 0.05 in the multivariate logistic regression were considered statistically significant and were weighted for the development of the IC score. For those regression coefficients that reached statistical significance in the model, each component was transformed into a subdomain score by dividing the coefficients by the smallest regression coefficient and then rounding up the absolute values of the coefficients to the nearest integer. Regression coefficients that were not statistically significant in the model were regarded as 1 point. A summary IC score was derived by adding points of all components. The higher the score was, the better the intrinsic capacity became.

For both the derivation and validation cohorts, model performance was evaluated by the area under the receiver operating curve (AUROC) and the Hosmer–Lemeshow goodness-of-fit test.

#### Phase 2

In the second phase of the study, we divided the study participants into three groups based on IC tertiles. The three groups were (1) High IC (Q3), (2) Medium IC (Q2), and Low IC (Q1). Continuous variables in the text and tables are expressed as the mean ± standard deviation, and categorical variables are expressed as percentages. Comparisons of baseline characteristics across different IC groups were performed by analysis of variance (ANOVA) for continuous variables and chi-square tests or Fisher's exact test for categorical variables. Ordinal logistic regression was used to examine the magnitude of association between biomarkers and IC groups, from low to high. Level of education, smoking status, socioeconomic status, and number of comorbidities were included as covariates in the adjusted model. Subgroup analysis was conducted in participants aged ≥60 years to evaluate whether the biological features of IC were different across age groups. The odds ratio (OR) and 95% confidential interval (95% CI) were reported.

#### Phase 3

In the last phase of the study, Kaplan–Meier survival analysis with the log-rank test was used to compare mortality risk between different IC groups. Cox proportional hazard models were used to explore the associations of IC score and 4-year all-cause mortality after adjustment for age, sex, levels of education, smoking status, socioeconomic status, number of comorbidities, and biomarkers. Kolmogorov-Type Supremum test was used to check the proportional hazards assumption for Cox proportional hazard models. We further performed subgroup analysis to assess the robustness of the results, and only participants aged 60 years and older were included. The hazard ratio (HR) and 95% confidential interval (95% CI) were reported.

Statistical significance was evaluated as *p* < 0.05, and all data were analyzed using SAS, version 9.4 (SAS Institute Inc., Cary, NC).

## Results

### Characteristics of the Study Subjects

The average age of 839 participants was 65.3 ± 9.4 years, and males accounted for the majority (54.1%) in the study. Among them, 15.9% did not receive any formal education (no schooling). The average number of comorbidities was 2.2 ± 1.8. The mean socioeconomic status among study subjects was level 4 (4.4 ± 1.8).

### Development and Validation of the Scoring System for Intrinsic Capacity

Multivariate logistic regression modeling in phase 1 for IC development involved 590 subjects (70%) in the derivation cohort and 249 subjects (30%) in the validation cohort. The final IC derivation model contained ten variables, five of which were statistically significantly associated with IADL impairments (*p* < 0.01). The significant regression coefficients of the variables in the IC model were transformed into integer subdomain scores, and the insignificant variables were counted as 1 point, as displayed in Table 1. Participants with robust chair stand speed and wellbeing had more capacity in terms of functional ability, weighted by two points; other subdomains were scored with one point. The summed IC score ranged from 0 to 12.

**Table 1:** Development and validation of scoring system for intrinsic capacity using multivariate logistic regression model.

**Intrinsic capacity (IC), 0–12**	**OR**	**95% CI**	***P*** **-value**	**Regression coefficient**	**Weight**
**Locomotion**					
**Gait speed**					
Slowness (Q1)	–	–	–	–	0
Robust (Q2–Q5)	0.42	0.23–0.76	<0.01	−0.87	1
**Chair stand**					
Slowness (Q1)	–	–	–	–	0
Robust (Q2–Q5)	0.19	0.11–0.35	<0.01	−1.64	2
**Sensory**					
**Visual acuity**					
With visual impairment	–	–	–	–	0
Without visual impairment	0.79	0.48–1.32	0.37	−0.23	1
**Hearing**					
With hearing loss	–	–	–	–	0
Without hearing loss	0.77	0.26–2.32	0.65	−0.26	1
**Vitality**					
**Body mass index**					
Malnutrition	–	–	–	–	0
Normal BMI	0.70	0.42–1.07	0.09	−0.40	1
**Grip strength**					
Weakness (male <28 kg, female <18 kg)	–	–	–	–	0
Robust (male ≧ 28 kg, female ≧ 18 kg)	0.32	0.19–0.53	<0.01	−1.15	1
**Psychological**					
**CES-D-10**					
Depression (>8)	–	–	–	–	0
Wellbeing (≦8)	0.22	0.12–0.39	<0.01	−1.52	2
**PSS-10**					
Higher level of stress (≧14)	–	–	–	–	0
Lower level of stress (<14)	0.61	0.36–1.03	0.06	−0.50	1
**Cognition**					
**SPMSQ**					
Cognitive impairment (≧3)	–	–	–	–	0
Cognitive health (<3)	0.33	0.15–0.71	<0.01	−1.11	1
**MMSE (language and recall)**					
Cognitive impairment (≦5)	–	–	–	–	0
Cognitive health (>5)	0.32	0.10–1.01	0.05	−1.13	1

*OR, Odds ratio; CI, confidence interval; BMI, Body mass index; CES-D-10, 10-item Center for Epidemiologic Studies Depression; PSS-10, 10-item Perceived Stress Scale; SPMSQ, Short Portable Mental State Questionnaire; MMSE, Mini-Mental State Examination*.

*AUROC: Derivation = 0.831, Validation = 0.858*.

*Hosmer-Lemeshow test: Derivation p = 0.173, Validation p = 0.143*.

The constructed IC model achieved AUROCs of 0.83 and 0.86 in the derivation and validation cohorts, respectively, confirming its good clinical performance in association with functional ability. The *p*-value for the Hosmer-Lemeshow test was 0.17 for the derivation cohort and 0.14 for the validation cohort, which was marginally calibrated but acceptable.

### Biomarkers

In the phase 2 study, participants were classified into a high IC group (11–12, *n* = 367), a medium IC group (9–10, *n* = 257), and a low IC group (0–8, *n* = 215) based on IC tertiles. Table 2 summarizes the baseline characteristics and biomarkers of the different IC groups. People in the low IC group were older, more likely to be women, have lower levels of education and had more comorbidities than those in the high and medium IC groups (*p* < 0.01). In addition, the low IC group had higher systolic blood pressure (*p* < 0.01) and serum levels of homocysteine (*p* < 0.01), IL-6 (*p* < 0.01), creatinine (*p* < 0.01), sICAM-1 (*p* < 0.01), hs-CRP (*p* < 0.01), urine epinephrine (*p* < 0.01), and allostatic load (*p* < 0.01) at baseline.

**Table 2:** Comparison of characteristics and biomarkers according to intrinsic capacity tertiles.

**Data values show M ±SD, or number (%)**	**Intrinsic capacity (IC) groups (*****n*** **=** **839)**			
	**High IC (11–12)**	**Medium IC (9–10)**	**Low IC (0–8)**	***P*** **-value**
Number	367	257	215	
Sex (female)	140 (38.2%)	120 (46.7%)	120 (55.8%)	<0.01
Age (year)	61.7 ± 7.5	64.6 ± 8.9	72.2 ± 9.0	<0.01
<60	191 (52.0%)	106 (41.2%)	30 (14.0%)	
60–64	70 (19.1%)	35 (13.6%)	13 (6.0%)	
65–74	72 (19.6%)	66 (25.7%)	73 (34.0%)	
75–84	30 (8.2%)	45 (17.5%)	79 (36.7%)	
≧85	4 (1.1%)	5 (2.0%)	20 (9.3%)	
The level of education				<0.01
No schooling	24 (6.5%)	41 (16.0%)	68 (31.6%)	
Elementary	161 (43.9%)	114 (44.3%)	92 (42.8%)	
Junior/senior high	114 (31.1%)	66 (25.7%)	45 (20.9%)	
College/graduate school	68 (18.5%)	36 (14.0%)	10 (4.7%)	
Smoking status				0.33
No	300 (81.7%)	201 (78.2%)	183 (85.1%)	
Sometimes	7 (1.9%)	5 (2.0%)	5 (2.3%)	
Frequently	60 (16.4%)	51 (19.8%)	27 (12.6%)	
Comorbidities^*^ (number)	1.6 ± 1.6	2.0 ± 1.8	3.2 ± 2.0	<0.01
Socioeconomic status	4.7 ± 1.7	4.3 ± 1.8	4.1 ± 1.8	<0.01
**INTRINSIC CAPACITY**				
**Locomotion**				
Gait speed (m/s)	0.9 ± 0.2	0.8 ± 0.2	0.6 ± 0.2	<0.01
Chair stand (s/5 stands)	8.7 ± 2.2	10.5 ± 4.1	15.3 ± 5.3	<0.01
**Sensory**				
Visual acuity	0.8 ± 0.3	0.6 ± 0.4	0.5 ±0.3	<0.01
Hearing loss	1 (0.3%)	5 (2.0%)	17 (7.9%)	<0.01
**Vitality**				
Body mass index (kg/m^2^)	24.4 ± 2.9	25.2 ± 3.5	25.4 ± 4.0	<0.01
Grip strength (kg)
Male	37.2 ± 7.2	33.1 ± 7.7	26.4 ± 8.3	<0.01
Female	22.8 ± 5.3	22.1 ± 12.5	15.7 ± 5.9	<0.01
**Psychological**				
CES-D-10 (0–30)	2.2 ± 2.5	4.4 ± 4.8	8.1 ± 6.7	<0.01
PSS-10 (0–40)	7.8 ± 5.1	10.7 ± 6.5	11.4 ±7.2	<0.01
**Cognition**				
SPMSQ (0–10)	1.2 ± 0.4	1.4 ± 0.7	1.9 ± 1.1	<0.01
MMSE (language and recall, 0–6)	6.0 ± 0.0	6.0 ± 0.2	5.8 ± 0.6	<0.01
**BIOMARKERS (*****N*** **=** **836)**				
**Cardiometabolic**				
Systolic blood pressure (mm Hg) (*n* = 838)	125.1 ± 18.8	129.0 ± 19.0	136.2 ± 21.6	<0.01
Diastolic blood pressure (mm Hg) (*n* = 838)	74.2 ± 10.1	72.9 ± 10.5	72.5 ± 11.1	0.13
Total cholesterol (mg/dL)	200.1 ± 38.5	200.5± 38.1	198.1 ± 37.9	0.77
HDL cholesterol (mg/dL)	47.7 ± 14.1	48.9 ± 13.4	47.7 ± 13.9	0.49
Triglycerides (mg/dL)	111.5 ± 67.4	112.1 ± 72.5	119.5 ± 71.6	0.37
Glycosylated hemoglobin (%)	6.0 ± 1.1	6.2 ± 1.2	6.3 ± 1.3	0.02
Fasting glucose (mg/dL)	103.9 ± 25.9	108.4 ± 32.0	110.5 ± 35.3	0.03
Waist-to-hip ratio (*n* = 838)	0.9 ± 0.1	0.9 ± 0.1	0.9 ± 0.1	<0.01
Homocysteine (μmol/L) (*n* = 828)	11.2 ± 4.7	11.3 ± 4.6	13.2 ± 5.6	<0.01
**Neuroendocrine**				
DHEA-S (μg/dL)	122.1 ± 75.9	114.6 ± 71.3	83.6 ± 60.0	<0.01
IGF-1 (nmol/L)	162.6 ± 64.9	147.6 ± 60.0	133.9 ± 56.8	0.04
Urine Cortisol (μg/g creatinine) (*n* = 813)	17.9 ± 27.9	18.7 ± 20.1	23.0 ± 45.5	0.15
Urine Epinephrine (μg/g creatinine) (*n* = 822)	3.9 ± 2.6	3.9 ± 2.3	4.6 ± 2.9	<0.01
Urine Norepinephrine (μg/g creatinine) (*n* = 822)	25.9 ± 12.2	27.5 ± 13.1	29.0 ± 16.4	0.03
Urine Dopamine (μg/g creatinine) (*n* = 822)	176.8 ± 66.0	184.1 ± 69.0	216.5 ± 623.6	0.34
**Inflammation and disease progression**				
White blood cell count (109 /L) (*n* = 826)	6.0 ± 1.7	6.1 ± 1.7	6.3 ± 1.8	0.04
Neutrophils (%) (*n* = 827)	57.0 ± 10.1	58.5 ± 9.3	58.9 ± 10.1	0.04
Interleukin-6 (pg/mL)	3.3 ± 6.8	3.3 ± 4.4	4.9 ± 7.6	<0.01
Creatinine (mg/dL)	0.9 ± 0.2	0.9 ± 0.3	1.1 ± 0.6	<0.01
sICAM-1(ng/ml)	254.2 ± 80.5	264.7 ± 84.9	291.6 ± 105.1	<0.01
Fibrinogen (mg/dL)	323.5 ± 61.5	330.0 ± 70.0	344.8 ± 70.3	0.01
hsCRP (mg/dl)	0.2 ± 0.3	0.3 ± 0.7	0.3 ± 0.7	<0.01
E-selectin (ng/mL)	38.3 ± 26.4	43.2 ± 30.7	44.2 ± 27.0	0.02
sIL-6R (ng/mL)	41.1 ± 11.4	43.1 ± 12.4	43.4 ± 13.0	0.04
Albumin (g/dL)	4.5 ± 0.3	4.5 ± 0.3	4.4 ± 0.3	<0.01
Folate (ng/mL)	9.4 ± 6.1	9.2 ± 5.5	10.1 ± 9.8	0.30
Allostatic load (0–12) (*n*= 797)	3.6 ± 2.1	3.7 ± 2.2	4.5 ± 2.3	<0.01
**GENETIC MARKERS**				
APOE ε4 carrier (*n* = 835)	49 (13.4%)	37 (14.6%)	38 (17.7%)	0.37
5-HTTLPR (*n* = 826)				0.63
S/S	167 (45.9%)	116 (46.2%)	93 (44.1%)	
S/L	128 (35.2%)	94 (37.5%)	86 (40.8%)	
S/XL	21 (5.8%)	17 (6.8%)	13 (6.2%)	
L/L or L/XL	48 (13.2%)	24 (9.6%)	19 (9.0%)	

*CES-D-10, 10-item Center for Epidemiologic Studies Depression; PSS-10, 10-item Perceived Stress Scale; SPMSQ, Short Portable Mental State Questionnaire; MMSE, Mini-Mental State Examination; DHEA-S, Dehydroepiandrosterone sulfate; IGF-1, Insulin-like growth factor-1; sICAM-1, Soluble intercellular adhesion molecule-1; hsCRP, high sensitivity C- reactive protein; sIL-6R, Soluble IL-6 receptor; APOE, Apolipoprotein E; 5-HTTLPR, The Serotonin Transporter Polymorphism*.

* *Comorbidities = self-report physician diagnosed chronic conditions, including hypertension, diabetes, heart disease, stroke, cancer, pulmonary disease, gastric disease, liver disease, arthritis, kidney disease, gout, cataract, degenerative joint disease, spinal/vertebrae spur, and hip fracture*.

**Figure 1:**
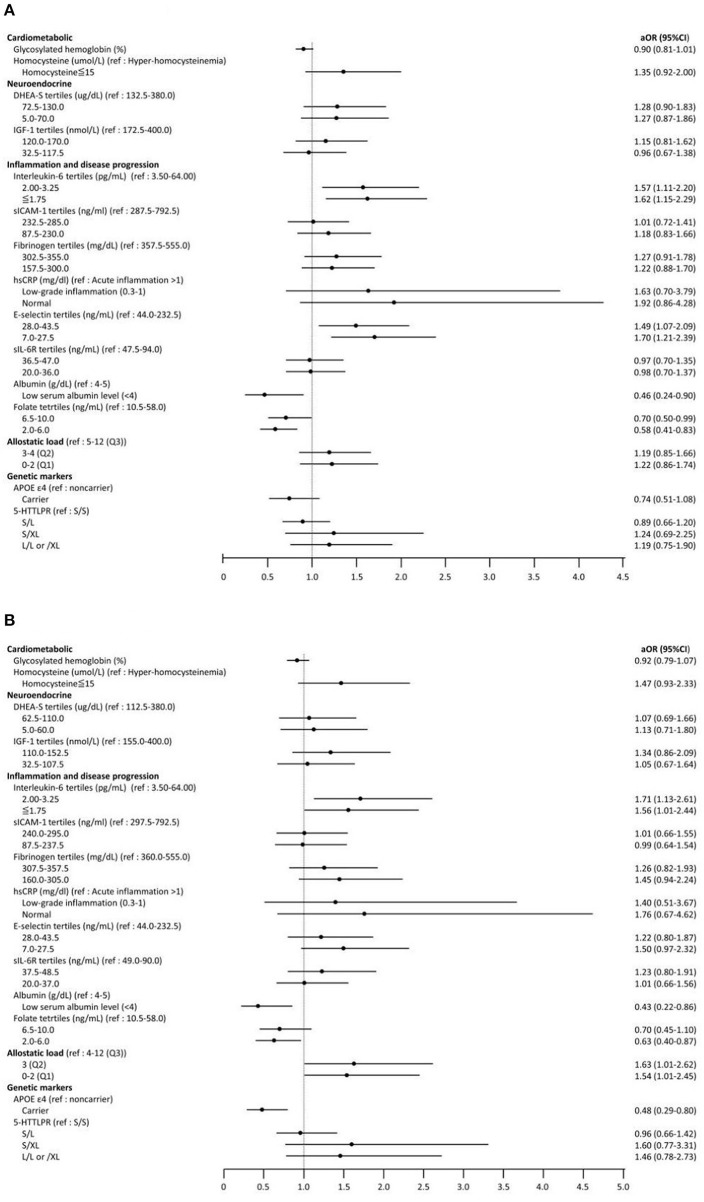
Forest plot for ordinary logistic regression examining the association between biomarkers and intrinsic capacity (IC) groups (from low IC to high IC). Adjust for age, sex, level of education, smoking status, socioeconomic status, and number of comorbidities. **(A)** Total study subjects (aged ≧ 50). **(B)** Subgroup analysis (aged ≧ 60). OR, Odds ratio; CI, Confidence interval; DHEA-S, Dehydroepiandrosterone sulfate; IGF-1, Insulin-like growth factor-1; sICAM-1, Soluble intercellular adhesion molecule-1; hsCRP, high sensitivity C- reactive protein; sIL-6R, Soluble IL-6 receptor; APOE, Apoliprotein E; 5-HTTLPR, The Serotonin Transporter Polymorphism.

The results of the ordinal logistic regression are presented in Figure 1 and Supplementary Table 1. After adjusting for age, sex, level of education, smoking status, socioeconomic status, and number of comorbidities, the associations between IC and numerous biomarkers remained significant. For people in the 1st ( ≤ 1.75 pg/ml) and 2nd tertiles (2.00–3.00 pg/ml) of serum IL-6 concentrations, the odds of being in the higher IC groups (i.e., high IC group or medium IC group vs. low IC group) were 62% (OR = 1.62; 95% CI, 1.15–2.29; *p* < 0.01) and 57% (OR = 1.57; 95% CI, 1.11–2.20; *p* < 0.01) higher than those of people in the 3rd tertile (3.50–64.00 pg/ml). For people in the lower tertiles of serum E-selectin concentration, the odds of being in higher IC groups were 70% (7.0–27.5 ng/ml: OR = 1.70; 95% CI, 1.21–2.39; *p* < 0.01) and 49% (28.0–43.5 ng/ml: OR = 1.49; 95% CI, 1.07–2.09; *p* = 0.01) higher than that of people in highest tertile of E-selectin concentration (44.0–232.5 ng/ml). Moreover, the odds of being in the higher IC group was 54% lower (OR = 0.46; 95% CI, 0.24–0.90; *p* = 0.01) in the low serum albumin level group (<4 g/dL) as compared with the higher albumin level group (4–5 g/dL) and 42% lower (2.0–6.0 ng/ml: OR = 0.58; 95% CI, 0.41–0.83; *p* < 0.01) in the lowest tertile of folate concentration compared to the highest tertile group (10.5–58.0 ng/ml) (Figure 1A).

In the subgroup analysis (Figure 1B), the associations between IL-6, albumin, folate and IC were similar to the main findings for all participants. In addition, compared to the reference group of AL (4–12 abnormal biomarkers), the odds of being in a higher IC group was 54% (OR = 1.54; 95% CI, 1.01–2.45; *p* = 0.04) higher for those with 0–2 abnormal biomarkers of AL. Notably, the presence of the APOE ε4 allele (OR = 0.48; 95% CI, 0.29–0.80; *p* < 0.01) was associated with being in a lower IC group only among people aged 60 years and older.

### Mortality

**Figure 2:**
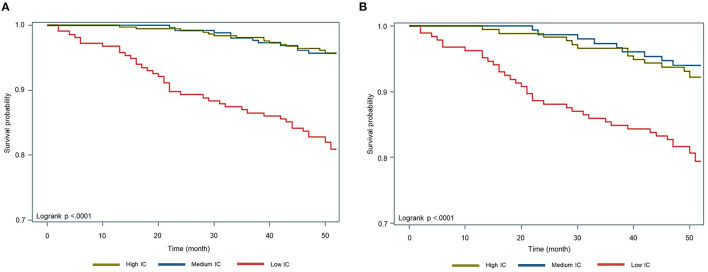
Kaplan-Meier survival curve by tertiles of intrinsic capacity. **(A)** Total study subjects (aged ≧ 50). **(B)** Subgroup analysis (aged ≧ 60).

Kaplan–Meier analysis showed significantly lower survival probability among the low IC group than the others (*p* < 0.01) (Figure 2). Table 3 shows the associations between IC groups and 4-year mortality. After adjustment for age, sex, level of education, smoking status, socioeconomic status, and the number of comorbidities, participants in the low IC group had a significantly higher mortality risk than people in the high IC group (HR = 3.02; 95% CI, 1.52–6.00; *p* < 0.01), whereas there was no statistical significance in the medium IC group (HR = 0.92; 95% CI, 0.42–2.03; *p* = 0.83). The results with additional adjustment for biomarkers persisted in the low IC group (HR = 2.50; 95% CI, 1.22–5.11; *p* = 0.01), as well as in the subgroup analysis (HR = 2.19; 95% CI, 1.03–4.64; *p* = 0.04).

**Table 3:** Tertiles of intrinsic capacity (IC) and 4-year mortality.

	**Unadjusted model**			**Model 1**			**Model 2**		
	**HR**	**95% CI**	***P*** **-value**	**HR**	**95% CI**	***P*** **-value**	**HR**	**95% CI**	***P*** **-value**
**Total study subjects (aged** **≧50)**									
High IC	*ref*	–	–	*Ref*	–	–	*ref*	–	–
Medium IC	1.05	0.48–2.28	0.91	0.92	0.42–2.03	0.83	0.84	0.38–1.88	0.67
Low IC	4.88	2.69–8.85	<0.01	3.02	1.52–6.00	<0.01	2.50	1.22–5.11	0.01
**Subgroup analysis (aged** **≧60)**									
High IC	*ref*	–	–	*ref*	–	–	*ref*	–	–
Medium IC	0.80	0.34–1.87	0.60	0.81	0.34–1.93	0.64	0.73	0.30–1.76	0.52
Low IC	2.89	1.53–5.45	<0.01	2.63	1.30–5.36	<0.01	2.19	1.03–4.64	0.04

*HR, Hazard ratio; CI, Confidence interval*.

*Model 1: adjust for age, sex, level of education, smoking status, socioeconomic status, and number of comorbidities*.

*Model 2: adjust for age, sex, level of education, smoking status, socioeconomic status, number of comorbidities and biomarkers (e.g., IL-6, E-selectin, albumin and folate for total study subjects; IL-6, albumin, folate and APOE gene for subgroup analysis)*.

## Discussion

To the best of our knowledge, this is the first study to develop an outcome-based IC scoring system and to examine its association with mortality. Moreover, this study also aimed to capture the biological features of IC to explore its potential underlying pathoaetiology and intervention strategies. In the present study, we found that serum levels of IL-6, E-selectin, serum albumin and folate were significantly associated with IC and that IC status substantially predicted 4-year mortality risk. However, among those aged 60 years and older, serum levels of IL-6, albumin, folate, allostatic load, and ApoE ε4 carriage were associated with IC status. Biomarkers associated with IC were more closely related to systemic inflammation, such as albumin, IL-6, and E-selectin, stress responses (allostatic load), and the ApoE ε4 genotype, which suggested potential mechanisms of aging across the lifespan.

In the context of healthy aging, this study used IADL impairments as the early loss of functional ability to weight each domain and develop an integrative IC score. The good performance of the ROAUC in both the derivation cohort and validation cohort indicates that the IC score is not only an empirically rigorous but also a useful assessment tool. In our scoring system, we found that the chair stand test (locomotion domain) and CES-D (psychological domain) accounted for higher weights (2 points), which implied the prognostic impacts of mobility and depression on overall functional ability. The results were compatible with previous studies in which the chair stand test was significantly associated with functional decline in a pooled analysis (34). In addition, poor mental health negatively affects the daily living of older people and increases the risk of ADL and IADL difficulties (35). Kondo et al. reported that old age depressive symptoms accelerated the deterioration of functional ability, particularly among old-old people (35). Therefore, more aggressive intervention programs focused on mobility and mental health are of critical importance to preserve intrinsic capacity, prevent loss of functional ability and promote healthy aging. In contrast to the frailty index developed by the cumulative deficit theory, the IC composite score in this study builds upon the construct of IC based on the WHO expert consensus and supporting evidence that shifted the focus from disease-centric to function-centric, and from reactive to preventive approaches. Although this study adopted incident IADL impairment as the outcome indicator to construct the IC composite score, the IC composite score was different from frailty index because the constructing domain of IC carried its own hypothesis in healthy aging and carried potentials for reversibility and improvement. The frailty index was constructed using various and sufficiently abundant amount of health data that substantially predicted adverse health outcomes, but the reversibility of frailty index was limited (36). Hence, the nature of IC composite score in this study was different from frailty index although both models were constructed to predict adverse outcomes.

Examining the associations between IC and biomarkers enabled us to justify the construction of the IC scoring system. Previous studies have demonstrated several promising biomarkers to predict frailty and other age-related syndromes (37–40). Frailty and IC are two constructs stemming from the same need to overcome traditional medical paradigms (41), and they may share common biological mechanisms and pathoaetiological processes. It has been reported that malnutrition and inflammation play critical roles in the pathophysiology of frailty (37–39). SEBAS examined multiple inflammatory biomarkers, such as c-reactive protein (CRP), interleukin-6 (IL-6), tumor necrosis factor-alpha (TNF-α), soluble intercellular cell adhesion molecule-1 (ICAM-1), and E-selectin. Our previous study revealed that ICAM-1 was independent of all other inflammatory biomarkers in predicting frailty using the same SEBAS dataset (42). However, in this study, based on the conceptual framework of IC, associations of inflammatory markers other than sICAM-1 were shown. Interestingly, the statistical significance of E-selectin disappeared when we focused on the older population (aged 60 years and older), but allostatic load and ApoE4 carriage became important factors. Compared to other inflammatory biomarkers, E-selectin was more strongly associated with endothelial dysfunction and subclinical atherosclerosis (43, 44), which may suggest that endothelial dysfunction started earlier in the aging process and was then followed by more systemic inflammatory responses. A recent study reported different roles of E-selectin and ICAM-1 in cardiac function over the life course of decades (45), so more in-depth studies are needed to clarify the different pathological roles of different inflammatory markers in the aging process. Recently, the roles of folate in aging and age-related diseases have attracted research attention because folate is an important dietary resource required to maintain and modulate DNA methylation (46).

Interestingly, this study only identified the associations between the ApoE4 allele and IC but not 5-HTTLPR. In the construct of the IC scoring system, only mobility and depressive symptoms were highly associated with IADL impairments. The discrepancy suggested potentially undiscovered confounding factors linking IC phenotypic presentation and genetic predisposition. Most likely, SPMSQ was not sensitive enough to identify early cognitive impairment, or ApoE4 was associated with multiple physiological functions in the aging process (47). Allostatic load, representing the dysregulated homeostasis of multiple organ systems from the accumulation of repeated or chronic stress (11, 40), was significantly associated with IC in this study, which indicated the roles of stress and related chronic systemic inflammation in the aging process.

Despite all of the efforts made in this study, there were still some limitations. First, the study sample size was relatively small, and therefore, the power of the study may be decreased. Second, we conducted a cross-sectional design to construct the IC scoring system based on the IADL impairments, which may not be able to establish causality between IC and functional impairments. Further longitudinal research may be warranted. In addition, we could only investigate potential biomarkers relevant to IC because of the lack of repeated measurements in follow-up studies. Third, the model performance on calibration in the current study was relatively marginal but acceptable. Future studies consider other variables that may be associated with the IC scoring system should be conducted to improve the model performance. Fourth, IC aims to capture early physiological changes in aging, so a longer follow-up period may be necessary to identify the long-term impacts of IC declines over time. Last but not the least, similar to most community aging cohort studies, the questionnaire and assessment tools aimed to capture “impairments” to predict adverse outcomes. However, this study used “not impaired” as the reference to describe the robustness of IC would underestimate the IC itself. Future research with different designs is needed to better capture the construct of IC.

In conclusion, the integrated IC scoring system, developed from the concept proposed by the WHO, substantially captured the mortality risk of people aged 50 years and older. The associations between IC and E-selectin, allostatic load and ApoE ε4 genotype suggested the underlying biological features of IC, indicating that mental health issues and endothelial dysfunction may be of greater impact in the biology of IC. Further investigations with a larger sample size and longer follow-up period are important to explore the longitudinal changes and impacts of IC in healthy aging.

## Data Availability Statement

The data analyzed in this study was obtained from the Social Environment and Biomarkers of Aging Study (SEBAS), the following licenses/restrictions apply: legal restrictions imposed by the government of Taiwan in relation to the Personal Information Protection Act. The public-use version is available to access (http://doi.org/10.3886/ICPSR03792.v7). Requests to access these datasets should be directed to ICPSR http://www.icpsr.umich.edu/icpsrweb/ICPSR/.

## Ethics Statement

The Institutional Review Board of Taiwan approved the study protocol, and written informed consents were obtained from all of the participants. The design and procedures of the study were carried out in accordance with the principles of the Declaration of Helsinki.

## Author Contributions

L-CM, S-TH, F-YH, and L-KC designed the research. L-CM, S-TH, L-NP, L-KC, and F-YH wrote the paper. L-CM analyzed data. F-YH and L-KC provided critical methodological inputs. L-CM, S-TH, and F-YH provided methodological and statistical inputs. L-NP and L-KC contributed to the clinical interpretation. All authors drafted the article, revised it critically for important intellectual content, and approved the final version to be published.

## Funding

This research was funded by the Ministry of Science and Technology, Taiwan (MOST-110-2634-F-010-001).

## Conflict of Interest

The authors declare that the research was conducted in the absence of any commercial or financial relationships that could be construed as a potential conflict of interest. The handling editor declared a past co-authorship with several of the authors L-KC and L-NP.

## Publisher's Note

All claims expressed in this article are solely those of the authors and do not necessarily represent those of their affiliated organizations, or those of the publisher, the editors and the reviewers. Any product that may be evaluated in this article, or claim that may be made by its manufacturer, is not guaranteed or endorsed by the publisher.
