# Supplementary material for: Biological Features of the Outcome-Based Intrinsic Capacity Composite Scores From a Population-Based Cohort Study: Pas de Deux of Biological and Functional Aging
Source: Front Med (Lausanne). 2022 Mar 4;9:851882. doi: 10.3389/fmed.2022.851882 (PMC8931213; doi:10.3389/fmed.2022.851882)
Supplement: Supplementary file 1 [file Table_1.DOCX]

Supplementary Material

**Supplementary Table 1. Ordinary logistic regression examining the association between biomarkers and intrinsic capacity(IC) groups (ordinal from low IC to high IC)**

|  | **Unadjusted Model** | | |  | **Model 1** | | |  | **Model 2** | | |
| --- | --- | --- | --- | --- | --- | --- | --- | --- | --- | --- | --- |
|  | OR | 95% CI | P Value |  | OR | 95% CI | P Value |  | OR | 95% CI | P Value |
| **Total study subjects (aged≧50)** |  |  |  |  |  |  |  |  |  |  |  |
| ***Biomarkers (****n=836)* |  |  |  |  |  |  |  |  |  |  |  |
| **Cardiometabolic** |  |  |  |  |  |  |  |  |  |  |  |
| Glycosylated hemoglobin (%) | 0.87 | 0.78-0.96 | <0.01 |  | 0.85 | 0.76-0.95 | <0.01 |  | 0.90 | 0.81-1.01 | 0.07 |
| Homocysteine (umol/L) **(**n=828) |  |  |  |  |  |  |  |  |  |  |  |
| Hyper-homocysteinemia (>15) | *ref* | *-* | *-* |  | *ref* | *-* | *-* |  | *ref* | *-* | *-* |
| ≦15 | 2.23 | 1.59-3.13 | <0.01 |  | 1.53 | 1.05-2.23 | 0.03 |  | 1.35 | 0.92-2.00 | 0.13 |
| **Neuroendocrine** |  |  |  |  |  |  |  |  |  |  |  |
| DHEA-S tertiles (ug/dL) |  |  |  |  |  |  |  |  |  |  |  |
| 132.5-380.0 | *ref* | *-* | *-* |  | *ref* | *-* | *-* |  | *ref* | *-* | *-* |
| 72.5-130.0 | 0.66 | 0.48-0.91 | 0.01 |  | 1.21 | 0.86-1.72 | 0.28 |  | 1.28 | 0.90-1.83 | 0.17 |
| 5.0-70.0 | 0.42 | 0.31-0.58 | <0.01 |  | 1.04 | 0.72-1.51 | 0.82 |  | 1.27 | 0.87-1.86 | 0.21 |
| IGF-1 tertiles (nmol/L) |  |  |  |  |  |  |  |  |  |  |  |
| 172.5-400.0 | *ref* | *-* | *-* |  | *ref* | *-* | *-* |  | *ref* | *-* | *-* |
| 120.0-170.0 | 0.79 | 0.57-1.08 | 0.13 |  | 1.21 | 0.87-1.69 | 0.26 |  | 1.15 | 0.81-1.62 | 0.43 |
| 32.5-117.5 | 0.44 | 0.32-0.60 | <0.01 |  | 0.94 | 0.66-1.34 | 0.75 |  | 0.96 | 0.67-1.38 | 0.83 |
| **Inflammation and disease progression** |  |  |  |  |  |  |  |  |  |  |  |
| Interleukin-6 tertiles (pg/mL) |  |  |  |  |  |  |  |  |  |  |  |
| 3.50-64.00 | *ref* | - | - |  | *ref* | - | - |  | *ref* | - | - |
| 2.00-3.25 | 2.00 | 1.45-2.75 | <0.01 |  | 1.63 | 1.17-2.28 | <0.01 |  | 1.57 | 1.11-2.20 | <0.01 |
| ≦1.75 | 2.92 | 2.13-4.00 | <0.01 |  | 1.91 | 1.36-2.66 | <0.01 |  | 1.62 | 1.15-2.29 | <0.01 |
| sICAM-1 tertiles (ng/ml) |  |  |  |  |  |  |  |  |  |  |  |
| 287.5-792.5 | *ref* | - | - |  | *ref* | - | - |  | *ref* | - | - |
| 232.5-285.0 | 1.33 | 0.98-1.81 | 0.07 |  | 1.13 | 0.81-1.56 | 0.47 |  | 1.01 | 0.72-1.41 | 0.96 |
| 87.5-230.0 | 2.07 | 1.51-2.84 | <0.01 |  | 1.49 | 1.07-2.08 | 0.02 |  | 1.18 | 0.83-1.66 | 0.36 |
| Fibrinogen tertiles (mg/dL) |  |  |  |  |  |  |  |  |  |  |  |
| 357.5-555.0 | *ref* | - | - |  | *ref* | - | - |  | *ref* | - | - |
| 302.5-355.0 | 1.36 | 0.99-1.86 | 0.05 |  | 1.27 | 0.91-1.76 | 0.15 |  | 1.27 | 0.91-1.78 | 0.17 |
| 157.5-300.0 | 1.52 | 1.12-2.06 | <0.01 |  | 1.23 | 0.89-1.70 | 0.22 |  | 1.22 | 0.88-1.70 | 0.24 |
| hsCRP (mg/dl) |  |  |  |  |  |  |  |  |  |  |  |
| Acute inflammation (>1) | *ref* | - | - |  | *ref* | - | - |  | *ref* | - | - |
| Low-grade inflammation (0.3-1) | 1.99 | 0.89-4.47 | 0.09 |  | 1.63 | 0.70-3.77 | 0.25 |  | 1.63 | 0.70-3.79 | 0.26 |
| Normal | 2.89 | 1.34-6.19 | <0.01 |  | 2.12 | 0.96-4.69 | 0.06 |  | 1.92 | 0.86-4.28 | 0.11 |
| E-selectin tertiles (ng/mL) |  |  |  |  |  |  |  |  |  |  |  |
| 44.0-232.5 | *ref* | - | - |  | *ref* | - | - |  | *ref* | - | - |
| 28.0-43.5 | 1.35 | 0.99-1.83 | 0.06 |  | 1.60 | 1.15-2.22 | <0.01 |  | 1.49 | 1.07-2.09 | 0.02 |
| 7.0-27.5 | 1.75 | 1.29-2.39 | <0.01 |  | 2.06 | 1.48-2.86 | <0.01 |  | 1.70 | 1.21-2.39 | <0.01 |
| sIL-6R tertiles (ng/mL) |  |  |  |  |  |  |  |  |  |  |  |
| 47.5-94.0 | *ref* | - | - |  | *ref* | - | - |  | *ref* | - | - |
| 36.5-47.0 | 1.17 | 0.91-1.70 | 0.32 |  | 1.02 | 0.74-1.41 | 0.90 |  | 0.97 | 0.70-1.35 | 0.85 |
| 20.0-36.0 | 1.25 | 0.86-1.59 | 0.16 |  | 1.11 | 0.80-1.54 | 0.54 |  | 0.98 | 0.70-1.37 | 0.92 |
| Albumin (g/dL) |  |  |  |  |  |  |  |  |  |  |  |
| 4-5 | *ref* | - | - |  | *ref* | - | - |  | *ref* | - | - |
| Low serum albumin level (<4) | 0.25 | 0.13-0.46 | <0.01 |  | 0.43 | 0.23-0.83 | 0.01 |  | 0.46 | 0.24-0.90 | 0.01 |
| Folate tertiles (ng/mL**)** |  |  |  |  |  |  |  |  |  |  |  |
| 10.5-58.0 | *ref* | - | - |  | *ref* | - | - |  | *ref* | - | - |
| 6.5-10.0 | 0.92 | 0.67-1.27 | 0.62 |  | 0.71 | 0.51-1.00 | 0.05 |  | 0.70 | 0.50-0.99 | 0.04 |
| 2.0-6.0 | 0.77 | 0.57-1.04 | 0.10 |  | 0.54 | 0.38-0.76 | <0.01 |  | 0.58 | 0.41-0.83 | <0.01 |
| **Allostatic load (n=797)** |  |  |  |  |  |  |  |  |  |  |  |
| 5-12 (Q3) | ref | - | - |  | ref | - | - |  | ref | - | - |
| 3-4 (Q2) | 1.21 | 0.88-1.64 | 0.24 |  | 1.21 | 0.87-1.67 | 0.26 |  | 1.19 | 0.85-1.66 | 0.31 |
| 0-2 (Q1) | 1.81 | 1.31-2.51 | <0.01 |  | 1.41 | 1.00-1.99 | 0.05 |  | 1.22 | 0.86-1.74 | 0.26 |
| ***Genetic markers*** |  |  |  |  |  |  |  |  |  |  |  |
| APOE ε4 (n=835) |  |  |  |  |  |  |  |  |  |  |  |
| noncarrier | *ref* | - | - |  | *ref* | - | - |  | *ref* | - | - |
| carrier | 0.79 | 0.55-1.12 | 0.18 |  | 0.68 | 0.47-0.99 | 0.04 |  | 0.74 | 0.51-1.08 | 0.12 |
| 5-HTTLPR (n=826) |  |  |  |  |  |  |  |  |  |  |  |
| S/S | *ref* | - | - |  | *ref* | - | - |  | *ref* | - | - |
| S/L | 0.87 | 0.66-1.16 | 0.34 |  | 0.88 | 0.66-1.19 | 0.41 |  | 0.89 | 0.66-1.20 | 0.44 |
| S/XL | 0.91 | 0.53-1.56 | 0.73 |  | 1.04 | 0.59-1.84 | 0.89 |  | 1.24 | 0.69-2.25 | 0.47 |
| L/L or L/XL | 1.35 | 0.88-2.08 | 0.18 |  | 1.20 | 0.76-1.90 | 0.43 |  | 1.19 | 0.75-1.90 | 0.46 |
| **Subgroup analysis (aged≧60)** |  |  |  |  |  |  |  |  |  |  |  |
| ***Biomarkers (****n=509)* |  |  |  |  |  |  |  |  |  |  |  |
| **Cardiometabolic** |  |  |  |  |  |  |  |  |  |  |  |
| Glycosylated hemoglobin (%) | 0.93 | 0.81-1.07 | 0.32 |  | 0.89 | 0.77-1.03 | 0.13 |  | 0.92 | 0.79-1.07 | 0.28 |
| Homocysteine (umol/L) **(**n=504) |  |  |  |  |  |  |  |  |  |  |  |
| Hyper-homocysteinemia (>15) | *ref* | *-* | *-* |  | *ref* | *-* | *-* |  | *ref* | *-* | *-* |
| ≦15 | 2.07 | 1.39-3.08 | <0.01 |  | 1.67 | 1.07-2.61 | 0.03 |  | 1.47 | 0.93-2.33 | 0.10 |
| **Neuroendocrine** |  |  |  |  |  |  |  |  |  |  |  |
| DHEA-S tertiles (ug/dL) |  |  |  |  |  |  |  |  |  |  |  |
| 112.5-380.0 | *ref* | *-* | *-* |  | *ref* | *-* | *-* |  | *ref* | *-* | *-* |
| 62.5-110.0 | 0.69 | 0.46-1.03 | 0.07 |  | 0.99 | 0.65-1.52 | 0.97 |  | 1.07 | 0.69-1.66 | 0.77 |
| 5.0-60.0 | 0.47 | 0.31-0.69 | <0.01 |  | 0.90 | 0.57-1.40 | 0.63 |  | 1.13 | 0.71-1.80 | 0.60 |
| IGF-1 tertiles (nmol/L) |  |  |  |  |  |  |  |  |  |  |  |
| 155.0-400.0 | *ref* | *-* | *-* |  | *ref* | *-* | *-* |  | *ref* | *-* | *-* |
| 110.0-152.5 | 1.12 | 0.75-1.66 | 0.59 |  | 1.63 | 1.07-2.50 | 0.02 |  | 1.34 | 0.86-2.09 | 0.20 |
| 32.5-107.5 | 0.61 | 0.41-0.91 | 0.01 |  | 1.09 | 0.71-1.68 | 0.68 |  | 1.05 | 0.67-1.64 | 0.83 |
| **Inflammation and disease progression** |  |  |  |  |  |  |  |  |  |  |  |
| Interleukin-6 tertiles (pg/mL) |  |  |  |  |  |  |  |  |  |  |  |
| 3.50-64.00 | ref | - | - |  | ref | - | - |  | ref | - | - |
| 2.00-3.25 | 1.88 | 1.27-2.77 | <0.01 |  | 1.72 | 1.14-2.59 | 0.01 |  | 1.71 | 1.13-2.61 | 0.01 |
| ≦1.75 | 2.53 | 1.69-3.79 | <0.01 |  | 1.77 | 1.15-2.73 | <0.01 |  | 1.56 | 1.01-2.44 | 0.04 |
| sICAM-1 tertiles(ng/ml) |  |  |  |  |  |  |  |  |  |  |  |
| 297.5-792.5 | *ref* | - | - |  | *ref* | - | - |  | *ref* | - | - |
| 240.0-295.0 | 1.11 | 0.75-1.65 | 0.60 |  | 1.11 | 0.73-1.69 | 0.62 |  | 1.01 | 0.66-1.55 | 0.98 |
| 87.5-237.5 | 1.64 | 1.11-2.44 | 0.01 |  | 1.19 | 0.78-1.81 | 0.42 |  | 0.99 | 0.64-1.54 | 0.97 |
| Fibrinogen tertiles (mg/dL) |  |  |  |  |  |  |  |  |  |  |  |
| 360.0-555.0 | *ref* | - | - |  | *ref* | - | - |  | *ref* | - | - |
| 307.5-357.5 | 1.36 | 0.92-2.02 | 0.12 |  | 1.26 | 0.83-1.91 | 0.28 |  | 1.26 | 0.82-1.93 | 0.30 |
| 160.0-305.0 | 1.69 | 1.14-2.51 | <0.01 |  | 1.47 | 0.97-2.24 | 0.07 |  | 1.45 | 0.94-2.24 | 0.09 |
| hsCRP (mg/dl) |  |  |  |  |  |  |  |  |  |  |  |
| Acute inflammation (>1) | *ref* | - | - |  | *ref* | - | - |  | *ref* | - | - |
| Low-grade inflammation (0.3-1) | 1.72 | 0.67-4.46 | 0.26 |  | 1.52 | 0.56-4.15 | 0.41 |  | 1.40 | 0.51-3.67 | 0.52 |
| Normal | 2.10 | 0.85-5.16 | 0.11 |  | 2.03 | 0.78-5.27 | 0.15 |  | 1.76 | 0.67-4.62 | 0.25 |
| E-selectin tertiles (ng/mL) |  |  |  |  |  |  |  |  |  |  |  |
| 44.0-232.5 | *ref* | - | - |  | *ref* | - | - |  | *ref* | - | - |
| 28.0-43.5 | 1.12 | 0.76-1.67 | 0.56 |  | 1.29 | 0.85-1.96 | 0.23 |  | 1.22 | 0.80-1.87 | 0.36 |
| 7.0-27.5 | 1.73 | 1.16-2.57 | <0.01 |  | 1.89 | 1.24-2.88 | <0.01 |  | 1.50 | 0.97-2.32 | 0.07 |
| sIL-6R tertiles (ng/mL) |  |  |  |  |  |  |  |  |  |  |  |
| 49.0-90.0 | *ref* | - | - |  | *ref* | - | - |  | *ref* | - | - |
| 37.5-48.5 | 1.42 | 0.95-2.13 | 0.09 |  | 1.33 | 0.87-2.05 | 0.19 |  | 1.23 | 0.80-1.91 | 0.35 |
| 20.0-37.0 | 1.16 | 0.78-1.72 | 0.45 |  | 1.13 | 0.74-1.72 | 0.58 |  | 1.01 | 0.66-1.56 | 0.97 |
| Albumin (g/dL) |  |  |  |  |  |  |  |  |  |  |  |
| 4-5 | *ref* | - | - |  | *ref* | - | - |  | *ref* | - | - |
| Low serum albumin level (<4) | 0.39 | 0.20-0.73 | <0.01 |  | 0.41 | 0.21-0.80 | <0.01 |  | 0.43 | 0.22-0.86 | 0.02 |
| Folate tertiles (ng/mL**)** |  |  |  |  |  |  |  |  |  |  |  |
| 10.5-58.0 | *ref* | - | - |  | *ref* | - | - |  | *ref* | - | - |
| 6.5-10.0 | 0.77 | 0.51-1.16 | 0.21 |  | 0.66 | 0.43-1.03 | 0.07 |  | 0.70 | 0.45-1.10 | 0.12 |
| 2.0-6.0 | 0.70 | 0.48-1.03 | 0.07 |  | 0.52 | 0.34-0.80 | <0.01 |  | 0.63 | 0.40-0.97 | 0.03 |
| **Allostatic load (n=482)** |  |  |  |  |  |  |  |  |  |  |  |
| 4-12 (Q3) | ref | - | - |  | ref | - | - |  | ref | - | - |
| 3 (Q2) | 1.53 | 0.99-2.36 | 0.06 |  | 1.64 | 1.03-2.61 | 0.04 |  | 1.63 | 1.01-2.62 | 0.05 |
| 0-2 (Q1) | 1.96 | 1.33-2.87 | <0.01 |  | 1.72 | 1.14-2.59 | <0.01 |  | 1.54 | 1.01-2.45 | 0.04 |
| ***Genetic markers*** |  |  |  |  |  |  |  |  |  |  |  |
| APOE ε4 (n=511) |  |  |  |  |  |  |  |  |  |  |  |
| noncarrier | *ref* | - | - |  | *ref* | - | - |  | *ref* | - | - |
| carrier | 0.60 | 0.38-0.94 | 0.03 |  | 0.46 | 0.28-0.76 | <0.01 |  | 0.48 | 0.29-0.80 | <0.01 |
| 5-HTTLPR (n=502) |  |  |  |  |  |  |  |  |  |  |  |
| S/S | *ref* | - | - |  | *ref* | - | - |  | *ref* | - | - |
| S/L | 0.94 | 0.66-1.34 | 0.73 |  | 0.94 | 0.65-1.37 | 0.75 |  | 0.96 | 0.66-1.42 | 0.85 |
| S/XL | 1.29 | 0.67-2.52 | 0.45 |  | 1.31 | 0.65-2.64 | 0.45 |  | 1.60 | 0.77-3.31 | 0.21 |
| L/L or L/XL | 1.71 | 0.97-3.02 | 0.07 |  | 1.55 | 0.85-2.84 | 0.15 |  | 1.46 | 0.78-2.73 | 0.23 |

OR=Odds ratio; CI=Confidence interval; DHEA-S= Dehydroepiandrosterone sulfate; IGF-1= Insulin-like growth factor-1; sICAM-1= Soluble intercellular adhesion molecule-1; hsCRP [=high sensitivity C- reactive protein; sIL-6R= Soluble IL-6 receptor; APOE =Apolipoprotein E; 5-HTTLPR=](http://www.labmed.org.tw/upfiles/magazinea/pdf/36-392103%20%20hscrp%EF%BC%88high%20sensitivity%20c-%20reactive%20protein%EF%BC%89%E7%9A%84%E8%87%A8%E5%BA%8A%E6%87%89%E7%94%A8%E5%9B%9E%E9%A1%A7%20%204.pdf)[The Serotonin Transporter Polymorphism](https://www.ncbi.nlm.nih.gov/pmc/articles/PMC2841212/)

[Model1 : Adjust for age and sex
Model2 : Adjust for age, sex, level of education, smoking status, socioeconomic status, and number of comorbidities](https://www.ncbi.nlm.nih.gov/pmc/articles/PMC2841212/)

**Supplementary Table 2. Cutoffs of Allostatic Load (AL) markers**

| **Allostatic load markers** | **M ± SD** | **Total Cut-off point (n=797)** | |  | **M ± SD** | **Subgroup Cut-off point (n=482)** | |
| --- | --- | --- | --- | --- | --- | --- | --- |
|  |  | **Low (<10%)** | **High (>90%)** |  |  | **Low (<10%)** | **High (>90%)** |
| **Cardiometabolic** |  |  |  |  |  |  |  |
| Systolic blood pressure (mm Hg) | 129.1 ± 20.2 | 104.0 | 157.3 |  | 134.6 ± 20.4 | 110.7 | 162.0 |
| Diastolic blood pressure (mm Hg) | 73.5 ± 10.4 | 61.3 | 88.0 |  | 75.6 ± 10.3 | 60.7 | 85.3 |
| Total cholesterol (mg/dL) | 200.4 ± 38.3 | 154.0 | 250.0 |  | 198.9 ± 37.7 | 154.0 | 248.0 |
| HDL cholesterol (mg/dL) | 47.9 ± 13.8 | 33.0 |  |  | 47.9 ± 13.7 | 34.0 |  |
| Triglycerides (mg/dL) | 114.9 ± 70.8 | 50 | 195 |  | 111.5 ± 64.7 | 50.0 | 195.0 |
| Glycosylated hemoglobin (%) | 6.1 ± 1.2 | 5.3 | 7.5 |  | 6.2 ± 1.1 | 5.3 | 7.5 |
| Fasting glucose (mg/dL) | 107.1 ± 30.6 | 86.0 | 140.0 |  | 107.3 ± 30.4 | 86.0 | 141.0 |
| Waist-to-hip ratio | 0.9 ± 0.1 | 0.8 | 1.0 |  | 0.9 ± 0.1 | 0.8 | 1.0 |
| Body mass index (kg/m^2^) | 24.9 ± 3.4 | 20.8 | 29.3 |  | 24.8 ± 3.5 | 20.6 | 29.1 |
| **Neuroendocrine** |  |  |  |  |  |  |  |
| DHEA-S (ug/dL) | 110.3 ± 72.5 | 30.0 |  |  | 91.1 ± 62.1 | 25.0 |  |
| IGF-1 (nmol/L) | 151.4 ± 62.2 | 77.5 | 235.0 |  | 136.4 ± 57.4 | 72.5 | 207.5 |
| Urine Cortisol (ug/g creatinine) | 19.5 ± 31.4 | - | 35.7 |  | 22.0 ± 39.2 | - | 38.6 |
| Urine Epinephrine (ug/g creatinine) | 4.0 ± 2.6 | - | 5.0 |  | 4.1± 2.6 | - | 4.4 |
| Urine Norepinephrine (ug/g creatinine) | 27.0 ± 13.6 | 13.5 | 43.8 |  | 27.1 ± 14.3 | 12.8 | 44.6 |
| Urine dopamine (ug/g creatinine) | 189.4 ± 322.1 | 104.2 | 263.2 |  | 188.0 ± 397.6 | 93.8 | 250.0 |
| **Inflammation and disease progression** |  |  |  |  |  |  |  |
| White blood cell count (10^9^ /L) | 6.1 ± 1.7 | 4.5 | 8.0 |  | 6.1 ± 1.7 | 4.5 | 8.0 |
| Neutrophils (%)(n=538) | 58.1 ± 9.6 | 46.0 | 69.0 |  | 59.0 ± 9.8 | 46.0 | 72.0 |
| Interleukin-6 (pg/mL) | 3.6 ± 6.3 | 1.0 | 5.8 |  | 3.8 ± 5.9 | 1.0 | 6.3 |
| Albumin (g/dL) | 4.4 ± 0.3 | 4.1 |  |  | 4.4 ± 0.3 | 4.0 |  |
| Creatinine (mg/dL) | 1.0 ± 0.4 |  | 1.3 |  | 1.0 ± 0.4 |  | 1.5 |

AL, allostatic load; DHEA-S, dehydroepiandrosterone sulfate; HDL, high density lipoprotein; IGF-1, insulin-like growth factor-1
